# Predictors of achieving minimal clinically important difference in functional status for elderly patients with degenerative lumbar spinal stenosis undergoing lumbar decompression and fusion surgery

**DOI:** 10.1186/s12893-024-02356-9

**Published:** 2024-02-16

**Authors:** Xiaofei Hou, Hailiang Hu, Peng Cui, Chao Kong, Wei Wang, Shibao Lu

**Affiliations:** 1 Department of Orthopaedics, Xuanwu Hospital, Capital Medical University, 45 Changchun Street, Xicheng District, Beijing, China; 2 Department of Orthopaedics, China National Clinical Research Center for Geriatric Disorders, Xuanwu Hospital, Capital Medical University, 45 Changchun Street, Xicheng District, Beijing, 100053 China

**Keywords:** Degenerative lumbar spinal stenosis, Functional status, Minimal clinically important difference, Predictors

## Abstract

**Objective:**

To identify the predictors for the achievement of minimal clinically important difference (MCID) in functional status among elderly patients with degenerative lumbar spinal stenosis (DLSS) undergoing lumbar decompression and fusion surgery.

**Methods:**

Patients who underwent lumbar surgery for DLSS and had a minimum of 1-year follow-up were included. The MCID achievement threshold for the Oswestry Disability Index (ODI) was set at 12.8. General patient information and the morphology of lumbar paraspinal muscles were evaluated using comparative analysis to identify influencing factors. Multiple regression models were employed to identify predictors associated with MCID achievement. A receiver operating characteristic (ROC) curve analysis was conducted to determine the optimal cut-off values for predicting functional recovery.

**Results:**

A total of 126 patients (46 males, 80 females; mean age 73.0 ± 5.9 years) were included. The overall rate of MCID achievement was 74.6%. Patients who achieved MCID had significantly higher psoas major muscle attenuation (43.55 vs. 39.23, *p* < 0.001) and preoperative ODI (51.5 vs. 41.6, *p* < 0.001). Logistic regression showed that elevated psoas major muscle attenuation (*p* = 0.001) and high preoperative ODI scores (*p* = 0.001) were independent MCID predictors. The optimal cut-off values for predicting MCID achievement were found to be 40.46 Hounsfield Units for psoas major muscle attenuation and 48.14% for preoperative ODI.

**Conclusion:**

Preoperative psoas major muscle attenuation and preoperative ODI were reliable predictors of achieving MCID in geriatric patients undergoing lumbar decompression and fusion surgery. These findings offer valuable insights for predicting surgical outcomes and guiding clinical decision-making in elderly patients.

**Supplementary Information:**

The online version contains supplementary material available at 10.1186/s12893-024-02356-9.

## Introduction

Degenerative lumbar spinal stenosis (DLSS) is a common cause of low back and leg pain in the elderly, and its incidence increases with age [1]. If conservative treatment fails, surgical treatment is often required [2]. While lumbar decompression and fusion surgery usually lead to positive outcomes, the extent of improvement can vary significantly among patients, especially those over 70 years old [3]. It’s crucial to identify factors influencing this variation to educate patients about their expected recovery, with a specific focus on achieving the minimal clinically important difference (MCID) in functional status.

The association between the morphology of paraspinal muscles and outcomes of lumbar spine surgery has recently been paid increasing attentions [4–8]. For instance, Zotti et al. [4] discovered that the preoperative cross-sectional area (CSA) of the multifidus muscle (MF) was a more reliable predictor of postoperative clinical outcomes, as measured by the Core Outcome Measures Index (COMI) and Oswestry Disability Index (ODI), in patients undergoing lumbar surgery. Similarly, Wang et al. [6] not only established a connection between the functional CSA as well as fat infiltration of the MF and preoperative ODI but also demonstrated their effectiveness as robust predictors for evaluating functional status improvements in DLSS patients. However, these studies mainly focused on lumbar extensor muscles. Given the accelerated degeneration of paraspinal muscles as individuals age [9], the potential of preoperative paraspinal muscle morphology in forecasting surgical outcomes for elderly DLSS patients remains unexplored.

The present study aimed to comprehensively examine the characteristics of paraspinal muscle in elderly patients with DLSS and explore whether degeneration of these muscle could predict the attainment of the MCID in the improvement of patients’ functional status.

## Methods

### Patients

After obtaining approval from the Ethical Committee of Xuanwu Hospital, patients over 65 years old admitted to the Department of Orthopaedics, Xuanwu Hospital who underwent lumbar decompression instrumented fusion for DLSS from February 2019 to December 2021 and had a minimum of 1-year follow-up were included. Additionally, we obtained informed consent from all patients who participated in this study.

The diagnostic criteria for DLSS were the presence of intermittent claudication and imaging features of spinal stenosis on lumbar MRI and CT. Patients with persistent symptoms and functional limitations despite conservative treatment were referred for surgery. The following patients were excluded: no available radiological findings within 2 years of surgery; with a history of previous lumbar spinal surgery; suffering from cachexia due to infectious diseases, cancer, myopathies, or dyskinesia were excluded.

### Outcome variable and predictors

The outcome variable was achieving MCID with a threshold of 12.8 (preoperative minus postoperative ODI) [10]. Patients were categorized into two groups at the final follow-up: those who achieved MCID and those who did not.

General information, including age, gender, body mass index (BMI), and preoperative ODI, were extracted from electronic medical records for each patient.

The preoperative Charlson comorbidity index was computed for each patient, serving as an assessment tool for baseline comorbidity burden and overall health status [11].

The Surgical invasiveness index was employed to assess the type and complexity of the surgical procedure [12].

The severity of postoperative complications was assessed using two metrics: the Clavien-Dindo classification system [13] and the Comprehensive complication index [14].

### Analytical morphometrics

**Fig. 1:**
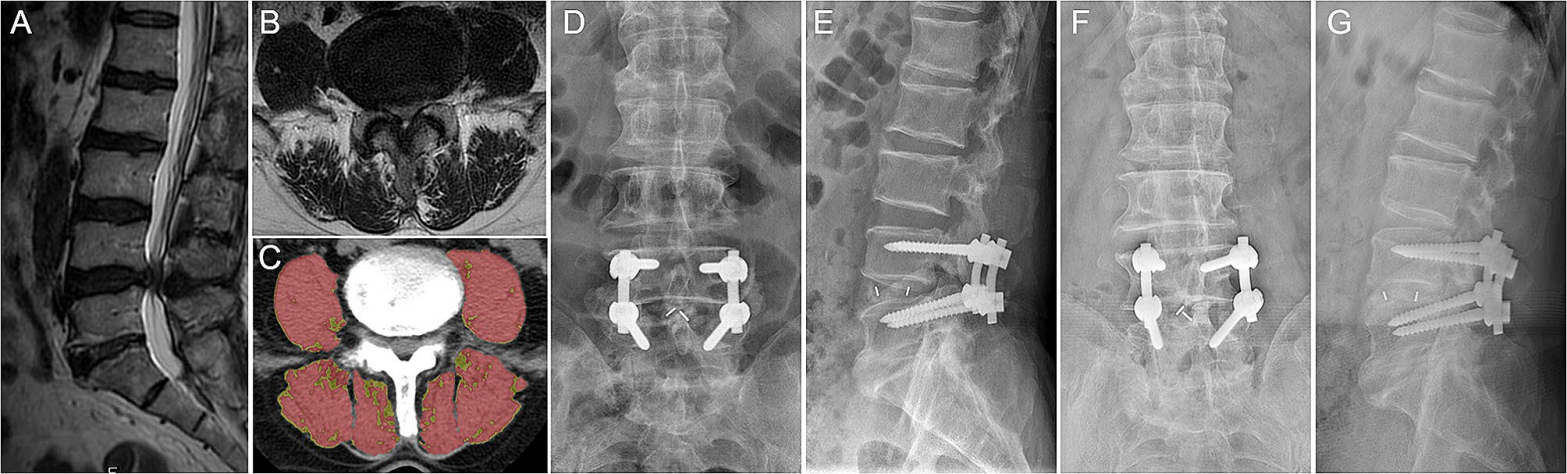
Radiographic data and measurement diagram of a 75-year-old male patient undergoing L4/5 decompression and fusion surgery for lumbar spinal stenosis. (**A**, **B**) Sagittal and axial MRI highlighting lumbar spinal stenosis at the L4/5 level. (**C**) CT image demonstrating the paraspinal muscle measurements. The muscle area is outlined in red, with CT values within the range of -29 to + 150. Average muscle attenuation corresponds to the mean CT value within the highlighted red area. (**D**, **E**) Anteroposterior and lateral radiographs of the lumbar spine 3 months post-surgery. (**F**, **G**) Anteroposterior and lateral radiographs of the lumbar spine captured 1 year after the operation

The morphometrics of paraspinal muscles were determined following established procedures [15, 16]. Specifically, an axial preoperative CT scan image aligned with the inferior vertebral endplate of L4 was imported into measurement software (AVW 2.0, Neusoft, Shenyang, China). When measuring the muscle area, we manually delineate the muscle contour along the fascial border to ensure accuracy. CSA for total lean multifidus muscle, erector spinae, and psoas major muscle were quantified within predefined validated boundaries of -29 to + 150 Hounsfield units, ensuring exclusion of non-muscular tissues [15]. All muscle areas were bilaterally measured at the inferior vertebral endplate of L4. The lean muscle area was standardized by the cross-sectional area of the intervertebral body at the same level. Average lean muscle attenuation was automatically calculated based on the outlined images (Fig. 1) [16].

In this study, muscular parameters were measured independently by two observers (XFH and HLH). The muscle data represent the average of measurements taken by the two observers. After a 1-week interval, the two observers repeated the measurements.

### Statistical analysis

Variables were compared between the two groups (MCID achieved and MCID not achieved) at the finial follow-up. Categorical variables were presented as counts and proportions. Continuous variables were assessed for normal distribution using the Kolmogorov–Smirnov test, and means with standard deviations (SDs) were used to describe those variables that exhibited a normal distribution. Univariate analysis was conducted using either the two independent samples t-test, Mann–Whitney U-test, chi-square test, or Fisher’s exact test, as deemed appropriate. Subsequently, multiple logistic regression analyses were employed, adjusting for all variables that approached statistical significance (with a p-value of < 0.1 in the univariate analysis) to identify independent predictors. The receiver operating characteristic (ROC) curve was employed to determine the optimal cutoff point, presenting the largest Youden index. The ROC curve was plotted using GraphPad Prism 5. Intraclass correlation coefficients (ICCs) were calculated to assess the intra- and inter-rater reliability for paraspinal muscle and vertebral body CSA, as well as muscle attenuation. All statistical analyses were performed using SPSS (version 28, IBM, Armonk, New York), with the criterion for statistical significance set at *p* ≤ 0.05.

## Results

### Descriptive data

126 patients were enrolled in the study, with an average age of 73.0 ± 5.9 years and a mean BMI of 25.0 ± 3.8 kg/m^2^. The majority of patients were female (63.5%). The overall MCID achievement rate was 74.6%. Patients who achieved MCID during postoperative follow-up exhibited significantly higher preoperative ODI scores but lower follow-up ODI scores (both *p* < 0.001). However, no notable differences in mean age, gender distribution, BMI, comorbidities, Charlson comorbidity index, Surgical invasiveness index, or Comprehensive complication index were observed between patients who achieved and those who did not achieve MCID. Further details are presented in Table 1.

**Table 1:** Baseline characteristics between patients who achieved and did not achieve MCID

	Overall	Achieved MCID	Did not achieve MCID	p value
No. of patients	126	94	32	
Age (in years)	73.0 ± 5.9	72.6 ± 6.0	74.1 ± 5.4	0.221
**Gender**				
-Female	80	58	22	0.192
-Male	46	36	10	
BMI (in kg/m^2^)	25.0 ± 3.8	24.8 ± 3.9	25.6 ± 3.6	0.301
Hypertension	76	55	21	0.477
Diabetes	47	37	10	0.412
Cardiovascular disease	26	17	9	0.225
Cerebrovascular disease	14	10	4	0.752^a^
Charlson comorbidity index (Mean ± SD)	1.0 ± 0.9	1.0 ± 0.9	1.0 ± 0.9	0.776
Surgical invasiveness index (Mean ± SD)	10.5 ± 3.3	10.2 ± 3.4	11.3 ± 2.5	0.112
Comprehensive complication index (Mean ± SD)	8.1 ± 11.4	7.9 ± 11.4	8.6 ± 11.7	0.768
Preoperative ODI	49.0 ± 13.6%	51.5%±11.8%	41.6%±15.8%	< 0.001
Finial followup ODI	18.7 ± 16.5%	12.1%±10.3%	38.0 ± 16.2%	< 0.001
Mean follow-up (in months)	17.9 ± 3.9	18.0 ± 4.1	17.8 ± 3.7	0.828

Fisher’s exact test. *Abbreviations* BMI, body mass index; MCID, minimal clinically important difference;ODI, Oswestry Disability Index

The most common reported complication was allogeneic blood transfusion (19.8%, *n* = 25), followed by delirium (5.6%, *n* = 7), nerve injury (4.0%, *n* = 5). Based on the Clavien-Dindo classification system, the majority of complications were categorized as grade II (85.4%). Supplementary Table 1 contains the types of the complications and their respective rates.

### Morphometric results

The intra-rater and inter-rater reliability of paraspinal muscle CSA, vertebral body CSA, and muscle attenuation showed high consistency, ranging from 0.849 to 0.977 (Table 2).

**Table 2:** Intra-rater and inter-rater reliability of paraspinal muscle parameters using intraclass correlation coeffecient

Measurements	Intra-rater ICC (95%CI)	Inter-rater ICC (95%CI)
Multifidus muscle CSA	0.901 (0.762–0.961)	0.865 (0.793–0.910)
Multifidus muscle attenuation	0.924 (0.816–0.970)	0.910 (0.874–0.936)
Erector spinae muscle CSA	0.885 (0.726–0.954)	0.849 (0.791–0.891)
Erector spinae muscle attenuation	0.916 (0.798–0.967)	0.914 (0.879–0.939)
Psoas major muscle CSA	0.962 (0.906–0.985)	0.952 (0.921–0.969)
Psoas major muscle attenuation	0.977 (0.942–0.991)	0.957 (0.938–0.970)
Vertebral CSA	0.934 (0.788–0.977)	0.921 (0.827–0.957)

*Abbreviations* CSA; cross-sectional area; CI, confidence interval; ICC, intraclass correlation coefficient

Among patients who achieved MCID, the average psoas major muscle attenuation was significantly higher than in those who did not achieve MCID (43.55 ± 6.31 vs. 39.23 ± 5.16, *p* < 0.001). Conversely, no significant difference was detected in the muscle CSA to vertebral CSA ratio and muscle attenuation in the multifidus and erector spinae muscles. Comprehensive details are available in Table 3.

**Table 3:** Morphometric measurements of paraspinal muscles

Measurements	Overall	Achieved MCID	Did not achieve MCID	p value
**Multifidus muscle**				
Muscle CSA / vertebral CSA	0.48 ± 0.13	0.48 ± 0.13	0.47 ± 0.10	0.671
Muscle attenuation	40.10 ± 12.15	40.38 ± 12.41	39.28 ± 11.52	0.660
**Erector spinae muscle**				
Muscle CSA / vertebral CSA	0.82 ± 0.19	0.81 ± 0.19	0.85 ± 0.21	0.416
Muscle attenuation	30.97 ± 11.02	31.08 ± 11.48	30.66 ± 9.71	0.853
**Psoas major muscle**				
Muscle CSA / vertebral CSA	0.90 ± 0.20	0.91 ± 0.20	0.88 ± 0.20	0.404
Muscle attenuation	42.45 ± 6.31	43.55 ± 6.31	39.23 ± 5.16	< 0.001

*Abbreviations* CSA; cross-sectional area; MCID, minimal clinically important difference

### Odds ratio and multivariate analysis of independent predictors for achievement of MCID

To determine the independent predictors associated with the achievement of MCID, both univariable and multivariable logistic regression analyses were conducted and the results are presented in Table 4. The psoas major muscle attenuation emerged as an independent predictor for MCID attainment, exhibiting an Odds Ratio (OR) of 1.141 (95% CI 1.054–1.236, *p* = 0.001). Similarly, preoperative ODI was identified as another independent predictor for MCID achievement, with an OR of 1.059 (95% CI 1.022–1.097, *p* = 0.001).

**Table 4:** Univariable and multivariable logistic regression analysis to identify independent predictors for achievement of MCID

Subject characters	Univariable analysis		Multivariable analysis	
	Odds Ratio, 95% CI	p value	Odds Ratio, 95% CI	p value
Male sex	1.366 (0.581, 3.212)	0.475		
Age	0.958 (0.895, 1.026)	0.221		
BMI	0.947 (0.854, 1.050)	0.300		
Charlson comorbidity index	0.938 (0.604, 1.455)	0.774		
Surgical invasiveness index	0.906 (0.802, 1.024)	0.115		
Comprehensive complication index	0.995 (0.961, 1.030)	0.766		
Preoperative ODI	1.055 (1.021, 1.091)	0.001	1.059 (1.022, 1.097)	0.001
**Psoas major muscle**				
Muscle CSA / vertebral CSA	2.436 (0.305, 19.434)	0.401		
Muscle attenuation	1.132 (1.050, 1.220)	0.001	1.141 (1.054, 1.236)	0.001
**Multifidus muscle**				
Muscle CSA / vertebral CSA	2.022 (0.081, 50.688)	0.668		
Muscle attenuation	1.007 (0.975, 1.041)	0.657		
**Erector spinae muscle**				
Muscle CSA / vertebral CSA	0.422 (0.053, 3.340)	0.413		
Muscle attenuation	1.004 (0.967, 1.041)	0.851		

*Abbreviations* BMI, body mass index; CI, confidence interval; CSA, cross-sectional area; ODI, Ostwestry Disability Index

**Fig. 2:**
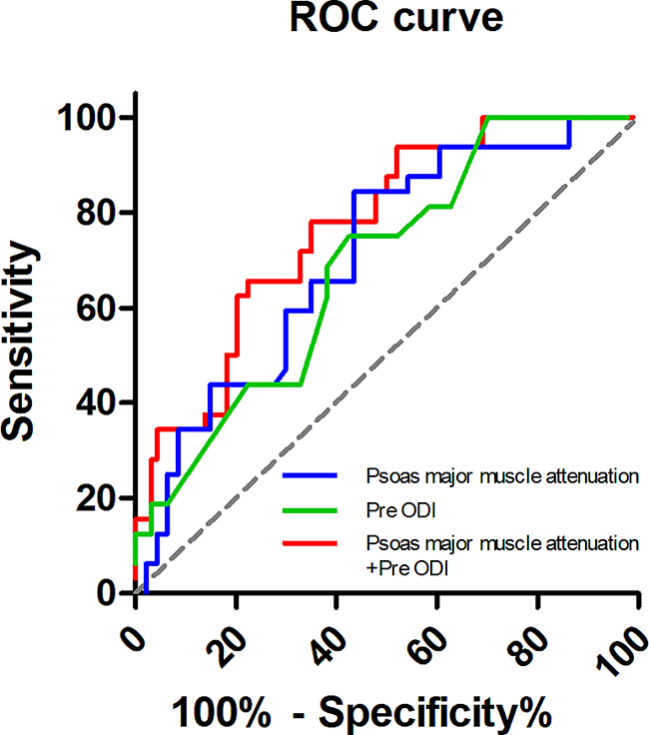
The receiver operating characteristic curves of the 3 predictors (psoas major muscle attenuation, preoperative ODI and psoas major muscle attenuation + preoperative ODI). *Abbreviations* ODI Ostwestry Disability Index

The determination of optimal cut-off values for psoas major muscle attenuation and preoperative ODI was achieved through the utilization of ROC curves and the calculation of the Youden index (Fig. 2; Table 5). The best cut-off value of psoas major muscle attenuation for predicting the achievement of MCID was 40.46 HU (AUC = 0.707, sensitivity = 0.702, specificity = 0.594). Likewise, the optimal cutoff value of preoperative ODI was 48.14% (AUC = 0.687, sensitivity = 0.617, specificity = 0.687). The AUC of psoas major muscle attenuation combined preoperative ODI was 0.770 (*p* < 0.001).

**Table 5:** The AUC in the ROC analysis

	AUC	95%CI	p value
Psoas major muscle attenuation	0.707	0.608–0.806	< 0.001
Preoperative ODI	0.687	0.587–0.786	0.002
Psoas major muscle attenuation + Preoperative ODI	0.770	0.682–0.858	< 0.001

*Abbreviations* AUC, area under the curve; ROC, receiver operating characteristic; CI, confidence interval; ODI, Ostwestry Disability Index

Subsequently, the predictive potential of preoperative ODI, muscle attenuation, and their combination was evaluated (Table 6). The combined muscle attenuation and preoperative ODI do not exhibit a more robust predictive capacity compared to each individual indicator (all *p* > 0.05).

**Table 6:** The comparison of predictive power of each factor

	δAUC	95%CI	p value
Psoas major muscle attenuation vs. preoperative ODI	0.021	−0.126–0.167	0.783
Psoas major muscle attenuation vs. Psoas major muscle attenuation + preoperative ODI	−0.063	−0.134–0.009	0.084
Preoperative ODI vs. psoas major muscle attenuation + Preoperative ODI	−0.083	−0.174–0.007	0.071

*Abbreviations* AUC, area under the curve; CI, confidence interval; ODI, Ostwestry Disability Index

## Discussion

Anticipating postoperative functional recovery following lumbar spine surgery is crucial for surgical planning. To best of our knowledge, this is the first study to investigate predictive factors associated with achieving MCID in elderly patients with DLSS after surgery. The current study revealed that 74.6% of patients achieved MCID in ODI after a follow-up period of at least 1 year. Higher preoperative psoas major muscle attenuation and ODI emerged as reliable predictors for attaining MCID in geriatric patients undergoing lumbar decompression and fusion surgery.

The psoas muscle plays a crucial role in supporting the anterolateral aspects of the lumbar spine, contributing significantly to lumbar stability [17]. This study suggests that elevated preoperative psoas major muscle density can serve as a predictive factor for achieving MCID following lumbar spine surgery. This association may be attributed to several factors: First of all, there is good consistency between psoas major muscle and the overall skeletal muscle mass. Therefore, patients with higher muscle attenuation of the psoas major muscle may have better overall nutritional reserve, which is beneficial for postoperative functional recovery [18, 19]; Secondly, the psoas major muscle plays a critical role for maintaining lumbar stability, and high quality muscles can provide local stability to promote lumbar fusion [20].

While the degeneration of lumbar extensor muscles, particularly the multifidus and erector spinae muscles, has been reported to influence the postoperative functional status of DLSS patients [4, 6], this study did not yield similar results. This discrepancy might be attributed to the remarkable degeneration of lumbar extensor muscles observed with increasing age, particularly in its distal end [21]. Given the advanced age of our study participants (mean 73.0 years old), the severe degeneration of the multifidus and erector spinae muscles might impede its predictive value for postoperative lumbar function.

This study has identified that a high preoperative ODI independently predicts the achievement of MCID after lumbar surgery. Similar finding was reported that there was a connection between high preoperative ODI and MCID achievement in patients undergoing minimally invasive lumbar decompression surgery [22]. Baseline ODI significantly influences the outcomes of lumbar spine surgery [23]. It’s reasonable to assume that patients with elevated preoperative disability, driven by the burden of their symptoms, are more likely to perceive a greater magnitude of improvement following surgery. Additionally, our study found a higher MCID achievement rate of 74.6%, compared to the reported 63.4% in the literature [22]. This could be attributed to the higher baseline ODI scores among our patients, potentially facilitating MCID attainment post-surgery.

In this study, although the AUC value for the combination of psoas major muscle attenuation and preoperative ODI surpasses the AUC values for each predictive indicator alone, there is no statistically significant difference in determining whether to achieve MCID after surgery. The possible reason for this may be due to the small sample size included in this study.

The findings of our study indicate that preoperative assessment of psoas major muscle attenuation emerges as a valuable tool to predict lumbar function recovery in elderly DLSS patients after surgery. This discovery underscores the significance of prehabilitation. Previous research has demonstrated that the quality of the psoas major muscle could be improved through an 8-week spinal stabilization exercise program [24, 25]. Therefore, a well-designed prehabilitation regimen encompassing physical exercises to bolster psoas major muscle quality prior to selective lumbar surgery may hold crucial clinical implications [26].

The research is subject to several limitations. To begin with, its retrospective cohort design inherently depends on the availability and quality of patient records within the database, influencing the level of evidence. Next, disparities may exist between reaching MCID and clinical improvement. However, when treatment effects meet the MCID threshold, it indicates their clinical significance and validates their application in clinical practice. Moreover, in this study, several patients with preoperative ODI < 20 were included. It is noteworthy that patients with low preoperative disability were less likely to achieve a significant reduction in their ODI meeting MCID criteria despite improvement after surgery. However, incorporating this subset of patients into the study may yield results that are more clinically meaningful. Additionally, the cutoff value for achieving MCID in postoperative ODI improvement was set at 12.8. While this value is commonly employed in the literature, it is not universally recognized as a gold standard. Further investigation is needed to determine whether muscle attenuation and preoperative ODI can still predict postoperative MCID when alternative cutoff values for MCID are applied. Furthermore, muscular measurements in this study were conducted using AVW software. Further research is needed to determine whether other muscle morphology assessment software, such as Image J, would yield similar conclusions. Lastly, the small sample size and single-center data in this study limit the generalizability of the findings. Future research endeavors should encompass larger, prospective, multicenter investigations to further validate the effectiveness and feasibility of employing psoas muscle attenuation and preoperative ODI scores in predicting the attainment of MCID in functional status for geriatric patients undergoing lumbar surgery.

## Conclusion

Identifying predictors for patients’ lumbar surgery outcomes is clinically crucial. This study indicates that preoperative psoas major muscle attenuation measured on lumbar CT scans and preoperative ODI can predict the attainment of MCID in lumbar function for elderly patients undergoing fusion surgery. Further validation of the study results is warranted through future multicenter, large-scale, prospective research.

### Electronic supplementary material

Below is the link to the electronic supplementary material.

**Supplementary Table 1.** Postoperative complications: frequency, CD grading and CCI score

## Data Availability

The datasets formed and assessed during this study can be obtained by reaching out to the corresponding author through a reasonable request.

## References

[1] Katz JN, Zimmerman ZE, Mass H, Makhni MC (2022). Diagnosis and management of lumbar spinal stenosis: a review. JAMA.

[2] Yoshihara H, Yoneoka D (2015). National trends in the surgical treatment for lumbar degenerative disc disease: United States, 2000 to 2009. Spine J.

[3] Alhaug OK, Dolatowski FC, Solberg TK, Lønne G (2023). Predictors for failure after surgery for lumbar spinal stenosis: a prospective observational study. Spine J.

[4] Zotti MGT, Boas FV, Clifton T, Piche M, Yoon WW, Freeman BJC (2017). Does pre-operative magnetic resonance imaging of the lumbar multifidus muscle predict clinical outcomes following lumbar spinal decompression for symptomatic spinal stenosis?. Eur Spine J.

[5] Chen J, Li J, Sheng B, Li L, Wu S (2022). Does preoperative morphology of multifidus influence the surgical outcomes of stand-alone lateral lumbar interbody fusion for lumbar spondylolisthesis?. Clin Neurol Neurosurg.

[6] Wang W, Sun Z, Li W, Chen Z (2020). The effect of paraspinal muscle on functional status and recovery in patients with lumbar spinal stenosis. J Orthop Surg Res.

[7] Han G, Zou D, Li X (2022). Can fat infiltration in the multifidus muscle be a predictor of postoperative symptoms and complications in patients undergoing lumbar fusion for degenerative lumbar spinal stenosis? A case-control study. J Orthop Surg Res.

[8] Amorim-Barbosa T, Catelas D, Pereira C (2023). Is preoperative fat infiltration in lumbar spine muscles associated with worse clinical outcomes after lumbar interbody fusion?. Eur J Orthop Surg Traumatol.

[9] Fortin M, Videman T, Gibbons LE, Battié MC (2014). Paraspinal muscle morphology and composition: a 15-yr longitudinal magnetic resonance imaging study. Med Sci Sports Exerc.

[10] Copay AG, Glassman SD, Subach BR, Berven S, Schuler TC, Carreon LY (2008). Minimum clinically important difference in lumbar spine surgery patients: a choice of methods using the Oswestry Disability Index, Medical outcomes Study questionnaire short form 36, and pain scales. Spine J.

[11] Charlson ME, Pompei P, Ales KL, MacKenzie CR (1987). A new method of classifying prognostic comorbidity in longitudinal studies: development and validation. J Chronic Dis.

[12] Mirza SK, Deyo RA, Heagerty PJ (2008). Development of an index to characterize the invasiveness of spine surgery: validation by comparison to blood loss and operative time. Spine (Phila Pa 1976).

[13] Dindo D, Demartines N, Clavien PA (2004). Classification of surgical complications: a new proposal with evaluation in a cohort of 6336 patients and results of a survey. Ann Surg.

[14] Slankamenac K, Nederlof N, Pessaux P (2014). The comprehensive complication index: a novel and more sensitive endpoint for assessing outcome and reducing sample size in randomized controlled trials. Ann Surg.

[15] Frontera WR, Ochala J (2015). Skeletal muscle: a brief review of structure and function. Calcif Tissue Int.

[16] Hicks GE, Simonsick EM, Harris TB (2005). Cross-sectional associations between trunk muscle composition, back pain, and physical function in the health, aging and body composition study. J Gerontol Biol Sci Med Sci.

[17] Penning L (2000). Psoas muscle and lumbar spine stability: a concept uniting existing controversies. Critical review and hypothesis. Eur Spine J.

[18] Hamaguchi Y, Kaido T, Okumura S (2016). Proposal for new diagnostic criteria for low skeletal muscle mass based on computed tomography imaging in Asian adults. Nutrition.

[19] Goodpaster BH, Carlson CL, Visser M (2001). Attenuation of skeletal muscle and strength in the elderly: the Health ABC Study. J Appl Physiol (1985).

[20] Wang W, Li W, Chen Z (2021). Risk factors for screw loosening in patients with adult degenerative scoliosis: the importance of paraspinal muscle degeneration. J Orthop Surg Res.

[21] Lee SH, Park SW, Kim YB, Nam TK, Lee YS (2017). The fatty degeneration of lumbar paraspinal muscles on computed tomography scan according to age and disc level. Spine J.

[22] Singh S, Shahi P, Asada T (2023). Poor muscle health and low preoperative ODI are independent predictors for slower achievement of MCID after minimally invasive decompression. Spine J.

[23] Pearson A, Lurie J, Tosteson T, Zhao W, Abdu W, Weinstein JN (2012). Who should have surgery for spinal stenosis? Treatment effect predictors in SPORT. Spine (Phila Pa 1976).

[24] Kim S, Kim H, Chung J (2014). Effects of spinal stabilization exercise on the cross-sectional areas of the lumbar multifidus and psoas major muscles, pain intensity, and lumbar muscle strength of patients with degenerative disc disease. J Phys Ther Sci.

[25] Owen PJ, Armbrecht G, Bansmann M (2020). Whey protein supplementation with vibration exercise ameliorates lumbar paraspinal muscle atrophy in prolonged bed rest. J Appl Physiol (1985).

[26] Nielsen PR, Jørgensen LD, Dahl B, Pedersen T, Tønnesen H (2010). Prehabilitation and early rehabilitation after spinal surgery: randomized clinical trial. Clin Rehabil.

