# Supplementary material for: Predictors of achieving minimal clinically important difference in functional status for elderly patients with degenerative lumbar spinal stenosis undergoing lumbar decompression and fusion surgery
Source: BMC Surg. 2024 Feb 16;24:59. doi: 10.1186/s12893-024-02356-9 (PMC10873985; doi:10.1186/s12893-024-02356-9)
Supplement: Supplementary file 1 — Supplementary Table 1. Postoperative complications: frequency, CD grading and CCI score [file 12893_2024_2356_MOESM1_ESM.docx]

Supplementary Table 1. Postoperative complications: frequency, CD grading and CCI score

| Frequency (%) | Complication | Procedures required | CD grade | CCI score |
| --- | --- | --- | --- | --- |
| 25 (19.8%) | Anemia | Allogeneic blood transfusion | II | 20.9 |
| 7 (5.6%) | Delirium | Medication | II | 20.9 |
| 5 (4.0%) | Nerve injury | Neurotrophic medicine ＆ rehabilitation training | II | 20.9 |
| 2 (1.6%) | Deep venous thrombosis | Anticoagulation | II | 20.9 |
| 2 (1.6%) | Atrial fibrillation | Anticoagulation | II | 20.9 |
| 4 (3.2%) | Superficial wound infection | Debridement ＆ antibiotics | III | 33.7 |
| 3 (2.4%) | Deep wound infection | Surgical revision ＆ antibiotics | III | 33.7 |

**Abbreviations:** CCI, comprehensive complication index; CD, Clavien-Dindo classification system.
